# The care cascade following a supportive management intervention for patients presenting to a radiation oncology clinic

**DOI:** 10.1038/s41598-022-27005-0

**Published:** 2022-12-29

**Authors:** Kaycee Moshofsky, Anastacia Aripov, Eric Chang, Michelle Bednar, Peter Bennett, Susan Hedlund, Kiri Cook

**Affiliations:** 1 Department of Pediatrics, University of Texas Southwestern Medical Center, Dallas, TX USA; 2 Children’s Health, Children’s Medical Center, Dallas, TX USA; 3 Department of Radiation Medicine, Oregon Health and Science University, Portland, OR USA; 4 Department of Radiation Oncology, Kaiser Permanente, Portland, OR USA

**Keywords:** Radiotherapy, Palliative care, Quality of life

## Abstract

Patients with cancer have many psychosocial needs, some of which may be addressed by implementation of a screening tool. However, it is unknown what ultimately happens (i.e., the “care cascade”) to patients following these interventions. The objective of this study was to evaluate the care cascade for patients following the implementation of a psychosocial needs screening tool. This was a prospective cohort study conducted at a university hospital radiation oncology clinic. Participants who were 18 years or older and presenting for their initial radiation oncology appointment were asked to complete a screening survey. From December 2019 to January 2021, 242 patients completed the survey. 70% of patients were seen for consideration of definitive therapy. 62% of patients checked “yes” to at least one item, most commonly supportive/palliative care (33%), exercise/PT (26%) and advance care planning (26%). Among definitive patients, the most common were supportive/palliative care (33%) and exercise/PT (26%). Among palliative patients, the most common were supportive/palliative care (42%) and advance care planning (32%). Participants were followed for 6 months after taking the survey. 74% of patients with a positive screening survey were contacted by a social worker and/or had a new referral placed with 47% of those patients ultimately attending a new appointment. Screening tools are commonly implemented to quickly identify needs in oncology patients. This study tracked patients following this type of intervention to determine what proportion of patients ultimately received care related to the identified need. Despite the majority of patients being referred to a relevant provider, fewer than half ultimately attended appointments. The combination of a screening tool with social work triage may be an effective way to distribute resources and properly route patients to supportive care providers.

## Introduction

Distress takes many forms, and it is commonly encountered during cancer care as a result of the diagnosis itself, effects of the disease, and consequences of the treatment. Cancer diagnosis and treatment has a pervasive impact on one’s life, including effects on physical and emotional health, alterations in social relationships, functional loss, and financial burden. Survey data demonstrates that 20–50% of patients with cancer experience significant distress during their disease course^1^. With 1,898,160 new cancer diagnoses anticipated in the United States in 2021, a significant number of people experience distress yearly secondary to cancer^2^.

The first step to addressing patient needs is identifying them, which should be included as a key component of comprehensive cancer care. Unfortunately, patient needs may go unrecognized for a variety of reasons including time constraints, provider discomfort, or patient embarrassment and fear. Patients may also be unaware of psychosocial support resources, particularly at the time of their first visit^3^. Screening tools can be used to help identify and address patient needs, including identifying those who would benefit from a referral to ancillary supportive care services^4,5^.

However, referral to ancillary services is only the first step to getting patients the care they need. To our knowledge, there are no published studies reporting on the cascade of care following these interventions in oncology patients. After a referral, how many patients are actually seen by ancillary providers? What are the barriers to doing so? In this study, we used a confidential tool to screen for sensitive patient needs in an attempt to identify the full range of concerns in our patient population. We followed patients who received the intervention to determine the impact the tool had on referrals, establishing care with SW, who often serves as a gateway to many supportive care resources, and ultimately, how many were seen by new providers. In addition, we surveyed providers before and after implementation of the tool to assess the impact on clinical workflow.

## Methods

### Study population

From December 2019 to January 2021, confidential screening checklists were distributed to patients presenting for initial consultation to an outpatient radiation oncology clinic. Participants were required to be 18 years or older. Patients were further separated into definitive or palliative cohorts. The initial consultation clinic note was retrospectively reviewed to collect whether radiation treatment was offered with a definitive or palliative intent to the patient being seen during their first clinic visit at the radiation oncology clinic.

### Screening checklist

An internally developed screening checklist was created to screen for needs that could then be acted upon by our care team. Therefore, the items chosen to be included on our checklist were known to be common amongst patients with cancer, but also those which aligned with our specific institutional resources. The checklist was delivered on paper, and asked patients to indicate “yes” or “no” to whether they would like more information about any of the following topics: supportive care (symptom and/or pain management), hospice or end of life care, advance care planning (advance directive, power of attorney), sexual health/intimacy, mental health (anxiety, depression, support groups), assistance at home (nursing, health aids), exercise or physical therapy, tobacco cessation, or drug or alcohol use. The checklist indicated that if the patient checked “yes” to any of the items, they would be contacted within 2–5 business days.

### Clinical procedure and data collection

Checklists were given to patients, along with intake paperwork, prior to being seen by a provider. The study was reviewed by the Oregon Health & Science University institutional review board (IRB) and was considered to be exempt from IRB oversight. Participation was voluntary and need for consent was waived. All methods were carried out in accordance with institutional guidelines and regulations. The areas of interest identified by the patient were either discussed during their appointment or followed up by social work/nursing within 2–5 business days. Additional data regarding patient demographics and medical history were obtained by review of their electronic health record. Patients were followed via chart review after the intervention to identify contact with social work, referrals placed, and new appointments attended.

### Provider surveys

Radiation Oncology physicians, residents, nurses, and medical assistants were invited to complete an anonymous survey in December 2019 prior to deployment of the screening tool. Providers were asked questions regarding the frequency at which they initiated conversations about sensitive topics with their patients, the importance of these conversations, their comfort level with these conversations, and barriers they experienced to addressing such topics. Providers were surveyed again in February 2020 to assess their comfort level with sensitive topic conversations, the impact of the survey on workflow and patient interactions, and if the survey helped address any barriers.

### Statistical analysis

Descriptive statistics were calculated to describe the patient population and summarize survey and follow-up data. Difference in rates of needs between treatment groups was tested using two-sample proportion tests. Hypotheses tests about differences for each need were tested using one sided proportion test without Yates’ continuity correction. Because of the small counts for tobacco cessation and drug/alcohol counseling, Fisher’s exact proportion test was used for testing proportions differences. Since the data have Poisson distribution, a negative binomial statistical model was used to test for difference in mean number of needs. For all results, p-values < 0.05 were considered statistically significant. Statistical analysis was done using RStudio V-1.4.1106 statistical software. GraphPad Prism 9 and RStudio were used to create all figures.

## Results

### Participant characteristics

Between December 2019 to January 2021, 1454 patients were seen for an in-person new patient consultation visit. Two hundred forty-two patients (16.6%) completed the screening checklist. Demographics of the patient population are summarized in Table 1. The average (SD) age for all participants is 61 (14) years, 58% are male, 80% are non-Hispanic, 53% are in a married/domestic partner relationship, and 53% are retired. Of the 242 patients, 70% of them were seen for a radiation therapy consultation with definitive treatment intent, while the other 30% were seen for consideration of palliative radiation therapy.

**Table 1:** Participant demographics. *SD* standard deviation.

	All participants (n = 242)	Definitive (n = 170)	Palliative (n = 72)
Average age (SD)	61 (14)	61 (15)	63 (12)
**Sex (%)**			
Male	141 (58)	102 (60)	39 (54)
Female	100 (41)	67 (39)	33 (46)
**Ethnicity (%)**			
Non-hispanic	193 (80)	139 (82)	54 (75)
Hispanic	15 (6)	10 (6)	5 (7)
Not listed	34 (14)	21 (12)	13 (18)
**Marital status (%)**			
Single	70 (29)	45 (27)	25 (35)
Married/domestic partner	128 (53)	90 (53)	38 (53)
Divorced/separated	22 (9)	16 (9)	6 (8)
Widowed	21 (9)	18 (11)	3 (4)
**Employment status (%)**			
Employed	71 (29)	60 (35)	11 (15)
Unemployed	26 (11)	15 (9)	11 (15)
Retired	129 (53)	84 (49)	45 (63)
Unknown	16 (7)	11 (7)	5 (7)
**Type of health insurance (%)**			
Medicare	102 (42)	69 (41)	33 (46)
Medicaid	50 (21)	34 (20)	16 (22)
Veteran	14 (6)	9 (5)	5 (7)
Private	76 (31)	58 (34)	18 (25)
**Advanced directive (%)**			
Yes	88 (36)	58 (34)	30 (42)
No	118 (49)	87 (51)	31 (43)
Unknown	36 (15)	25 (15)	11 (15)

Factors related to participants’ health status and diagnosis are reported in Table 2. The most common disease sites were hematologic (17%), gastrointestinal (15%), and prostate (14%). The reason for relatively lower numbers of other common disease sites (i.e., breast) is the distribution of visit locations at our institution; most breast patients are seen in a multidisciplinary clinic at a different site. Most participants (78%) have at least one chronic comorbidity. The most common comorbidities are hypertension (53%), mental health diagnosis (42%), and cardiovascular disease (23%). Fifty-four percent of participants reported having a smoking history, while fewer participants (14%) reported current tobacco use.

**Table 2:** Participant health information. *BMI* body mass index, *SD* standard deviation, *COPD* chronic obstructive pulmonary disease, *WHO* World Health Organization, *CNS* central nervous system.

	All participants (n = 242)	Definitive (n = 170)	Palliative (n = 72)
BMI (average, SD)	29 (6)	29 (7)	29 (6)
**Tobacco use n (%)**			
Current smoker	33 (14)	21 (12)	12 (17)
Smoking history	130 (54)	92 (54)	38 (53)
**Alcohol use n (%)**			
Current user	95 (39)	76 (45)	19 (26)
History of alcohol abuse	15 (6)	10 (6)	5 (7)
**Comorbidities n (%)**			
Cardiovascular disease	56 (23)	38 (22)	18 (25)
Hypertension	128 (53)	88 (52)	40 (56)
Diabetes	43 (18)	28 (17)	15 (21)
Liver disease	28 (12)	18 (11)	10 (14)
COPD	25 (10)	16 (9)	9 (13)
Chronic kidney disease	23 (10)	12 (7)	11 (15)
Prior malignancy	51 (21)	38 (22)	13 (18)
Mental health diagnosis	101 (42)	75 (44)	28 (39)
**Karnofsky performance status**			
Median (range)	90 (30–100)	90 (60–100)	80 (30–100)
Unknown (%)	47 (19)	33 (19)	15 (21)
**Stage n (%)**			
0	8 (3)	8 (5)	0
1	32 (13)	31 (18)	1 (1)
2	27 (11)	26 (15)	1 (1)
3	40 (17)	38 (22)	2 (3)
4	80 (33)	22 (13)	58 (81)
Hematologic	42 (17)	32 (19)	10 (14)
WHO I–IV	13 (5)	13 (8)	0
**Primary malignancy disease site n (%)**			
Hematologic	41 (17)	32 (19)	9 (13)
Gastrointestinal	37 (15)	19 (11)	18 (25)
Prostate	33 (14)	27 (16)	6 (8)
Lung	28 (12)	13 (8)	15 (21)
Head/neck	23 (10)	20 (12)	3 (4)
CNS	21 (9)	18 (11)	3 (4)
Gynecologic	17 (7)	15 (9)	2 (3)
Melanoma	12 (5)	9 (5)	3 (4)
Breast	9 (4)	4 (2)	5 (7)
Sarcoma	6 (3)	4 (2)	2 (3)
Non-melanoma skin cancer	4 (2)	4 (2)	0
Mycosis fungoides	4 (2)	1 (1)	3 (4)
Non-prostate genitourinary	3 (1)	1 (1)	2 (3)
Other	4 (2)	3 (2)	1 (1)

### Participant reported needs

**Figure 1:**
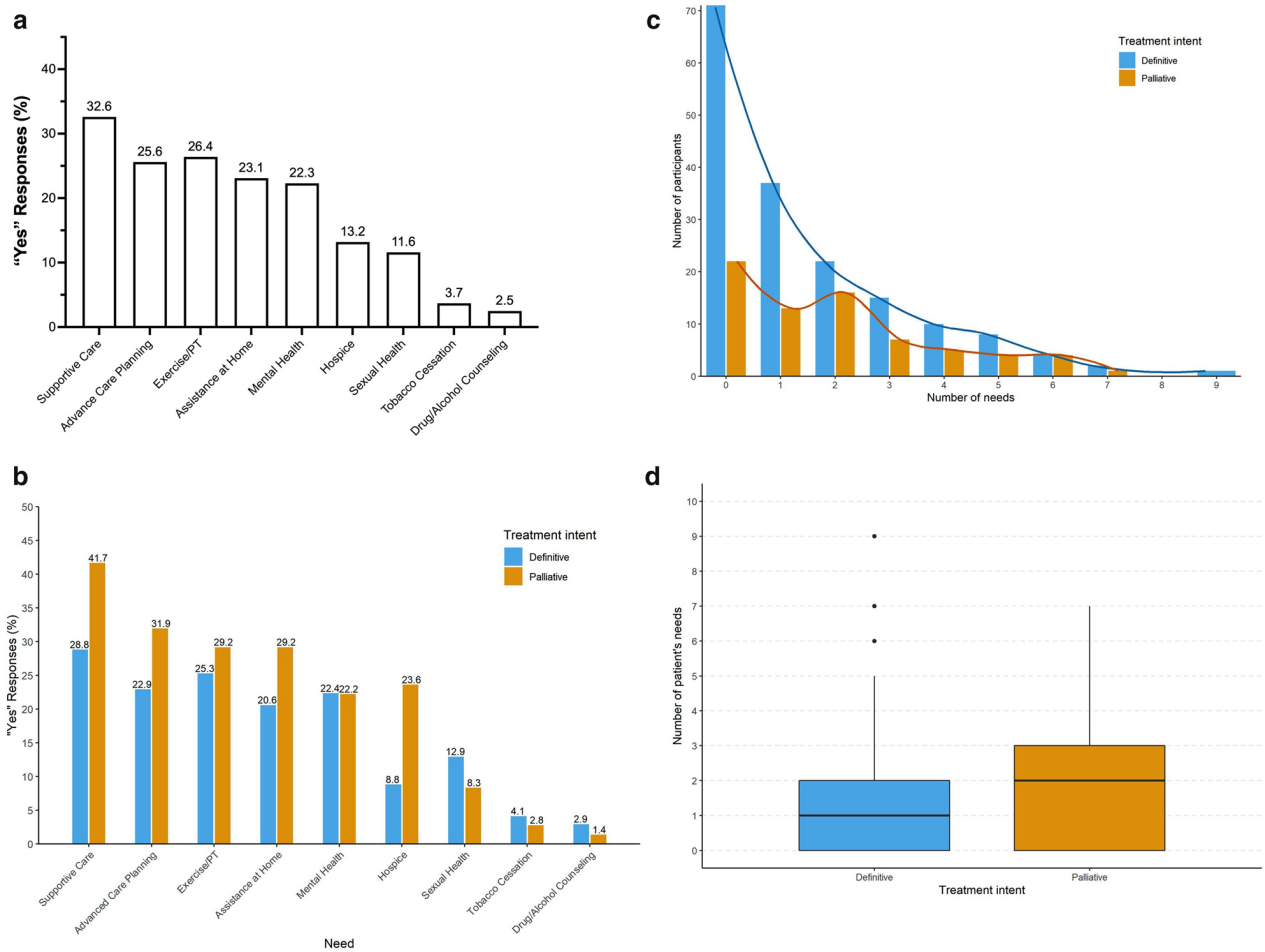
Summary of patient needs. Summary of identified survey needs for (**a**) all participants and (**b**) by treatment intent. (**c**) Number and (**d**) distribution of the number of patients’ needs by treatment intent. Based on negative-binomial statistical model, there is no evidence for difference in mean number of needs between the two groups of participants (p-value = 0.143).

Sixty-two percent of participants checked “yes” to at least one item on the checklist with the specific distribution of needs displayed in Fig. 1a. Patient needs were separated by treatment intent (Fig. 1b). Sixty-nine percent of patients in the palliative treatment group identified at least one need on the survey compared to 58% of patients in the definitive treatment group. However, a two-sample proportion test revealed no significant difference in the proportion of patients who identified at least one need between the two groups (p-value = 0.1, CI [− 0.24 to 0.018]).

**Figure 2:**
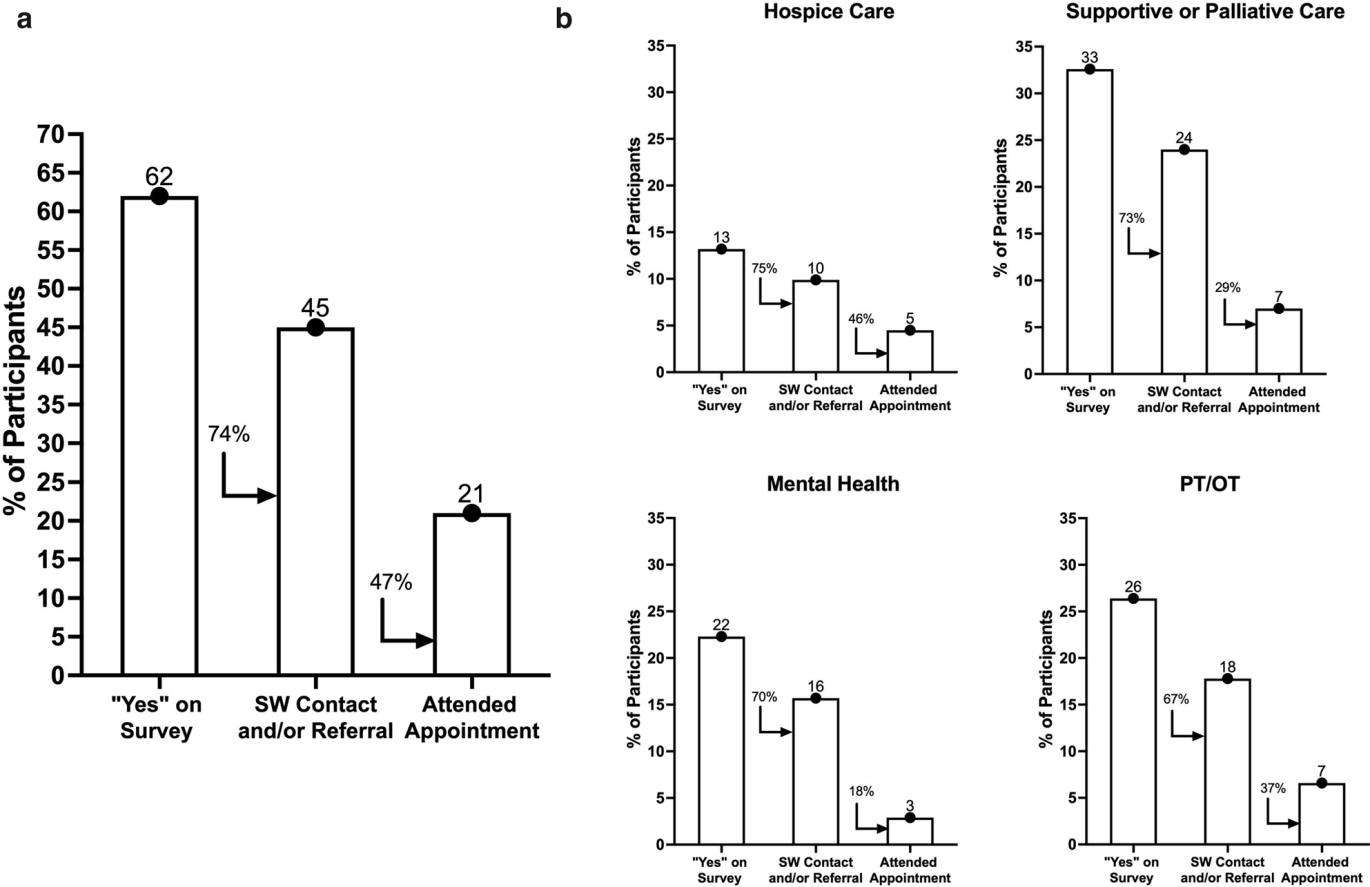
Care cascade after intervention. Summary of the progression of care after a positive response on a screening survey for (**a**) all participants and (**b**) for specific responses.

Overall, the most commonly identified needs were supportive care (33%), exercise/PT (26%), and advance care planning (26%) (Fig. 1a). For the palliative intent group, the most commonly checked items included supportive care (42%), advance care planning (32%), assistance at home (29%), and exercise/PT (29%) (Fig. 2b). For the definitive intent group, the most common items were similar and included supportive care (29%), exercise/PT (25%), advance care planning (23%), and mental health (22%). Tests of equal proportionality were performed to evaluate for differences in specific needs between treatment groups. Table 3 demonstrates that palliative patients request information about supportive care and hospice care more often than definitive patients (p-values = 0.026 and 0.0001, respectively). In addition, the palliative group requested information more often about advance care planning and assistance at home, although the difference is not statistically significant (p-values = 0.071 and 0.074, respectively). Additionally, Table 3 highlights that there is no evidence for significant difference in proportions of patients asking for mental health, exercise/PT, sexual health, tobacco cessation and drug/alcohol counseling resources.

**Table 3:** Summary of patient needs. The number of patients asking for information about tobacco cessation and drug/alcohol counseling is very small in both groups, which limits statistical testing.

Need	% Definitive patients	% Palliative patients	p-value	Confidence interval
Supportive care	28.8	41.7	0.026	(− 1, − 0.017)
Advance care planning	22.9	31.9	0.071	(− 1, 0.015)
Exercise/PT	25.3	29.2	0.266	(− 1, 0.065)
Assistance at home	20.6	29.2	0.074	(− 1, 0.016)
Hospice	8.8	23.6	< 0.001	(− 1, − 0.058)
Mental health	22.4	22.2	0.982	(− 0.133, 0.116)
Sexual health	12.9	8.3	0.306	(− 0.035, 0.127)
Tobacco cessation*	4.1	2.8	1	(0.276, 15.16)
Drug/alcohol counseling*	2.9	1.4	0.673	(0.234, 103.13)

*There were very few patients in each group requesting information on tobacco cessation and drug/alcohol counseling resources, limiting the validity of statistical testing in this population.

Participants checked yes to an average of 1.6 survey items. Patients undergoing consultation for palliative treatment checked more items on average (1.9), compared to those with definitive intent (1.5). The distribution of the number of needs is displayed in Fig. 1c,d. Based on a negative-binomial statistical model, there is no evidence for a difference in mean number of needs between the two groups of participants (p-value = 0.143).

### Survey follow-up and impacts

Participants were followed by chart review after the intervention to identify contact with social work, referrals made and new provider appointments attended. The median follow-up was 6 months. Of the 62% of patients who identified one need on the survey, 74% were contacted by SW and/or had a referral placed. Of those, 47% established care with a new provider that they were not previously seeing prior to taking the survey (Fig. 2a). Of those with 1 or more referrals placed, 45% of the referrals were for palliative care or for pain management, 43% for PT/OT, and 17% for hospice care. Additional data on this care cascade for some of the most commonly identified needs, including hospice care, supportive or palliative care, mental health, and PT or OT, are displayed in Fig. 2b.

### Provider survey feedback

Prior to the implementation of the patient screening survey, a survey was sent to 24 providers, including 2 medical assistants, 6 nurses, 6 resident physicians, and 10 attending physicians. Twenty staff members completed the pre-implementation survey. Every provider who was surveyed felt that addressing sensitive topics with patients was very or extremely important. Most providers (65%) indicated that patients did not frequently initiate discussions about sensitive topics. Providers reported several barriers that inhibited their ability to address these topics with their patients, with the most commonly reported barrier being “not enough time.” The post-implementation survey was completed by 20 participants. About half (45%) of survey respondents, including 67% of attending physicians, reported having more conversations with patients about sensitive topics after checklist implementation. Fifty-five percent of providers, including 78% of attending physicians, felt that the screening survey helped alleviate the “not enough time” barrier. Half of the providers felt that they made more referrals after implementation, while the other half reported no impact on referrals. While 88% of attending physicians and 60% of residents said there was no impact on workflow, 83% of nurses/MAs responded workflow was “a little harder”.

## Discussion

It is considered standard of care to screen patients with cancer for distress, at least at the initial consultation^1^, however many providers report that they infrequently engage in conversations with patients about challenging topics such as mental health, sexual health, or advance care planning. This is likely multifactorial, but commonly cited reasons are a lack of time and either provider or patient reluctance to initiate conversations about difficult topics. Busy clinics and external pressure can limit the amount of time physicians spend with their patients^6^, and most of that time is typically spent discussing diagnosis and treatment^7^. Unsurprisingly, shorter clinic visits are associated with less attention spent to patients’ psychosocial problems, and decreased referrals to ancillary support^8,9^.

Realistically, clinic visit slots are unlikely to lengthen in the foreseeable future, but holistic care of the individual remains a critical piece of oncologic care. A screening checklist is a valuable tool to rapidly identify sensitive needs. Clinicians report that screening tools help to improve rapport and identify problems that may otherwise not have been discussed^10^. Additionally, patients tend to view screening tools favorably and consider them important to their care^10,11^. Through a large systematic review and meta-analysis, Faller et al. demonstrated that a variety of psycho-oncologic interventions such as these improve distress symptoms and quality of life^12^. Additionally, identifying and addressing patient needs has been shown to have positive effects on health, including reducing symptoms, alleviating worry, and improving outcomes^13–15^.

In this study, a high percentage (62%) of patients identified at least one need on the screening checklist, whether they were presenting for definitive or palliative management. While patients presenting for palliative radiation were more likely to check “yes” to a few specific needs, a large number of patients presenting for definitive management also requested information on needs including supportive care, advance care planning, and assistance at home. In addition to providing a window into what topics may be important to our patient population, surveyed providers indicated that this checklist resulted in more conversations about those topics as well as more referrals to ancillary providers. Specifically, it was felt that the survey helped alleviate the “not enough time” barrier many providers reported experiencing. Because a “yes” response on the checklist results in nursing/social work intervention, this enables needs to be addressed even when the physician does not have time to discuss them at length.

In this study, we followed patients to determine the care cascade after the supportive care intervention. We demonstrated that a large fraction (74%) of participants with a positive survey were contacted by social work and/or had a new referral placed after the completion of the screening survey. However, less than half of patients then went on to attend a new appointment. There may be several reasons for this drop-off. For instance, patients may later decline a referral after further education, or they may elect for an intervention not requiring a referral; for example, a patient chooses to join a support group instead of attending an appointment with a mental health provider. Patients may also benefit from hearing from social work about available referrals, but decide to wait to engage these services at a later date. Regardless of ultimate appointment attendance, the screening survey opened a line of communication through which patients can be educated about resources and referrals of which they may have previously been unaware. Additionally, it is important to consider the time constraints of other providers, such as palliative care and mental health physicians, and that it may not be feasible or even beneficial to automatically refer all patients to all of the services they indicate on the survey.

This study has limitations. A non-validated, internally developed screening checklist was used for this study in order to tailor the screening questions to include institutional specific resources, however this may limit the external validity and generalizability of our study results. While we attempted to identify a broad range of needs, it is unlikely that we have a comprehensive list. Some needs, while they may be important to patients, are more challenging to address with referrals within the medical center such as religious or cultural concerns, and these were not included. Additionally, though there were nearly 250 survey respondents, these numbers are still too small to make more granular conclusions such as what specific needs may be important to patients with one particular disease site over another. Future studies will attempt to include a longer follow-up period to report on downstream utilization of resources, further assessing for reasons for drop-out in the care cascade, and assess whether certain demographics or disease sites correlate with specific needs or higher susceptibility to drop-out.

In conclusion, screening checklists are a quick, inexpensive, noninvasive tool to identify sensitive patient needs in the outpatient oncology setting. Screening surveys can help identify needs of which patients may have been unaware and create an environment supportive of requesting additional information on these difficult topics. This study demonstrates that a large proportion of patients, regardless of treatment intent, have a variety of psychosocial needs that are important to screen for. We demonstrated that a survey which screens for needs that can be addressed by existing institutional resources can both prompt valuable conversations with providers as well as facilitate direct access to ancillary services without provider oversight. Routing requests for information through social work may be an efficient way to connect patients with the providers they need without overburdening ancillary support services. Further refinement of the tool may help to avoid it negatively impacting clinical workflow.

## Data Availability

Raw data can be made available upon request to the corresponding author.
